# Trauma-Related Clinical Practice Variation in Dutch Emergency Departments

**DOI:** 10.3390/healthcare11050748

**Published:** 2023-03-03

**Authors:** Elise L. Tierie, Dennis G. Barten, Laura M. Esteve Cuevas, Rebekka Veugelers, Menno I. Gaakeer

**Affiliations:** 1 Department of Emergency Medicine, Albert Schweitzer Hospital, 3318 AT Dordrecht, The Netherlands; 2 Department of Emergency Medicine, Admiraal de Ruyter Hospital, 4462 RA Goes, The Netherlands; 3 Department of Emergency Medicine, VieCuri Medical Centre, 5912 BL Venlo, The Netherlands

**Keywords:** practice variation, clinical variation, The Netherlands, emergency medicine, benchmark

## Abstract

Structural insights in the use of protocols and the extent of practice variation in EDs are lacking. The objective is to determine the extent of practice variation in EDs in The Netherlands, based on specified common practices. We performed a comparative study on Dutch EDs that employed emergency physicians to determine practice variation. Data on practices were collected via a questionnaire. Fifty-two EDs across The Netherlands were included. Thrombosis prophylaxis was prescribed for below-knee plaster immobilization in 27% of EDs. Vitamin C was prescribed in 50% of EDs after a wrist fracture. Splitting of applied casts to the upper or lower limb was performed in one-third of the EDs. Analysis of the cervical spine after trauma was performed by the NEXUS criteria (69%), the Canadian C-spine Rule (17%) or otherwise. The imaging modality for cervical spine trauma in adults was a CT scan (98%). The cast used for scaphoid fractures was divided between the short arm cast (46%) and the navicular cast (54%). Locoregional anaesthesia for femoral fractures was applied in 54% of the EDs. EDs in The Netherlands showed considerable practice variation in treatments among the subjects studied. Further research is warranted to gain a full understanding of the variation in practice in EDs and the potential to improve quality and efficiency.

## 1. Introduction

Emergency departments (EDs) in The Netherlands use a variety of guidelines to set up protocols for diagnostics modalities and treatments. Initially, the majority of these protocols were set up locally [1,2]. At present, 51 multidisciplinary medical guidelines from various organisations and specialties are endorsed by The Netherlands Society of Emergency Physicians (NSEP/NVSHA). Nine of these guidelines were initiated and/or developed by the NSEP [3].

All faculties of the current EDs in the Netherlands have access to these (inter)national guidelines. Their use creates a platform for standardisation in emergency medicine. Nonetheless, medical practice variation may still occur since variation in clinical practice is seen in numerous departments and countries, including the Netherlands [4,5,6,7].

Although the guidelines are nationally available, structural insights in the use of protocols and the extent of practice variation in emergency medicine are lacking [8].

Some degree of variation is not unjustified and to some extent variation is necessary, because patients/situations are different [9]. Nonetheless, apparent diversity cannot solely be attributed to the variations in patient presentation or preference of a physician [6]. Such variation may originate from the complexity of healthcare environments, novel insights, lack of valid clinical knowledge, ambiguity towards the evidence base, financial conflicts or overreliance on subjective judgement [7,9,10,11]. It illustrates that unwarranted clinical variation is a complex matter [12].

Practice variation can lead to an increase in costs and suboptimal results, consequently being a motive for further healthcare development [7,10,13]. “Addressing variations in care supports the triple bottom line—improved quality, increased efficiency, and a better patient experience [10]”.

By standardising protocols, healthcare can be homogenised to streamline diagnoses and treatments. The basic principles of such standardisation have a voluntary base, are an open process, require consensus and should create compatibility between hospitals [4]. By applying these principles, insight in practice variation could lead to a more targeted optimisation policy in the future and may benefit the development of emergency medicine as a whole [14,15]. Therefore, it would be valuable to perform a comparative study to signal different patterns of practice between hospitals [6].

## 2. Materials and Methods

In 2020–2021, we performed a comparative study by questionnaire that is best described as a cross-sectional survey. The study population consisted of EDs in The Netherlands that were included if the ED is hospital-based, open 24/7 and employs board-certified emergency physicians. The Netherlands has 79 listed EDs, of which 12 were primarily excluded because they do not employ board-certified emergency physicians.

The data were collected via a questionnaire set up by several emergency physicians who are experts in their field. The questionnaire contained multiple choice questions of a nominal or scale character (Table A1 and Table A2 in Appendix A). All input from the questionnaire was saved to a single database, where input was structured per (anonymous) hospital. Recruitment was exclusively performed through medical managers of the EDs, who are also emergency physicians, by e-mail. The medical manager was asked to fill out the questionnaire on behalf of their department with answers based on local protocol or generally acknowledged common practice. Departments participated voluntarily and were not offered a monetary incentive. After six weeks, a simultaneous digital notice was sent out to all EDs as a reminder for participation and the final deadline one week later.

Since most national guidelines concern trauma-related conditions, the questioned practices in this study are linked to trauma. The seven included practices are: thrombosis prophylaxis for lower limb immobilisation, fracture-related vitamin C prescription, splitting of applied casts for upper and lower limbs, cervical spine imaging, wound suturing, plaster choice in case of scaphoid fracture and the application of a Fascia Iliaca Compartment Block (FICB). These practices were chosen because they are pragmatic, generally low in complexity and often have a binary character. The guidelines and protocols by the Dutch National Federation of Medical Specialists (FMS) and other national specialist databases were consulted in July and August of 2021. Furthermore, general ED characteristics of the study participants were collected and subsequently analysed to provide possible context for the findings.

The study parameters were computed from the database using Microsoft Excel 2016 and expressed in a percentage of EDs that practise a specified protocol.

## 3. Results

In this study, 67 EDs spread across The Netherlands were approached to participate. Of these, 15 were excluded because there was no response to the request to participate, resulting in 52 included EDs in this study (76%). The descriptive statistics of the included EDs are depicted in Table 1. The results of the questionnaire are presented in Table 2.

### 3.1. Thrombosis Prophylaxis

Thrombosis prophylaxis was prescribed by default for below-knee plaster immobilisation in 27% of the EDs, whilst in 8% of the EDs thrombosis prophylaxis was never prescribed for this type of immobilisation. In 21% of EDs, the PADUA score was used and other factors were applied in the remaining 44% (Caprini score, local guidelines, pre-existent thromboembolism risk factors in medical history, the Dutch Quality Institute for Healthcare (CBO) guideline or depending on the emergency physician on duty).

For the above-knee plaster immobilisation, 75% of EDs prescribed it by default, 8% prescribed it based on the PADUA score and 17% based on other factors.

In total, 71% of the EDs did not prescribe thrombosis prophylaxes for the immobilisation of the lower extremity by means of a knee brace or temporary splint, and 12% of the EDs prescribed it by default, 4% based on the PADUA score and 13% otherwise.

The most frequently used anticoagulation was nadroparine (66%), followed by dalteparine (35%), tinzaparine (2%) and other forms of low-molecular-weight heparin (LMWH) (2%). Dosages were uniform for all patients (38%) or based on body mass index (BMI) (44%). The remainder based dosages on the patient’s weight, the Caprini score, kidney function or on local hospital guidelines.

### 3.2. Vitamin C

Vitamin C was prescribed by default in 27% of EDs after a distal wrist fracture, 58% never prescribed vitamin C and 15% only after a repositioning of the wrist fracture. Of the EDs that prescribed vitamin C, it was mostly prescribed from the age of 18+ years (59%), but other age limits included 16 years (11%), ≥40 years (4%) or ≥50 years (26%). All EDs except for one prescribed a dose of 500 mg per day. The prescription period ranged from 2 weeks (4%) to 4 weeks (4%), 7 weeks (7%), 50 days (57%), 2 months (4%) and 3 months (14%), or only during the period of the immobilisation of the fracture (11%).

### 3.3. Splitting of Cast

Splitting of lower or upper limb casts was performed by 31% of the EDs. About half of the EDs noted that their department no longer applies circular casts for lower limbs and instead works with plaster splints.

### 3.4. Cervical Spine Trauma

The majority of EDs assess the cervical spine after trauma using the NEXUS criteria (69%). Other EDs use the Canadian C-spine Rule (17%), a combination of the two decision rules, the guideline by the FMS or physician’s preference. The primary imaging modality for cervical spine trauma in adults was computerized tomography (CT) scan (98%). The other 2% primarily perform an X-ray of the cervical spine.

### 3.5. Wound Suture Circumstances

Sterile suture sets were used by default in 90% of EDs. Sterile gloves when suturing a wound were used in 31% of EDs, sterile drapes in 17% and none of the above in 12%. A few departments noted that it differs per physician. Wounds were cleaned prior to suturing with one of several solutions. The most commonly used solution was sterile water (73%), followed by tap water (25%), Hibicet: a mix of chlorhexidine and cetrimide (62%), sodium chloride (8%) and a mix of lidocaine and cetrimide (2%).

### 3.6. Scaphoid Fracture

The type of cast used for scaphoid fractures was fairly divided between the short arm cast (46%) and the navicular cast (54%).

### 3.7. FICB

The most commonly used method was the ultrasound-guided FICB (40%). In none of the EDs were FICB blocks solely placed based on the anatomic landmarks of a patient. In total, 12% of EDs performed a femoral block instead of an FICB and 2% a pericapsular nerve group (PENG) block. In 4% of EDs, locoregional anaesthesia was performed by anaesthesiologists.

## 4. Discussion

Our study shows that practice variation is widespread among Dutch EDs. The results showed that practice variation was greatest in thromboprophylaxis, vitamin C prescriptions, splitting of casts and scaphoid fracture treatment. The clinical diagnostic tool for cervical spine injury and the use of ultrasound for FICB was found to be fairly uniform across all EDs.

Research on the effectiveness of thromboprophylaxis generally indicates that pharmacological prophylaxis could decrease overall venous thromboembolism (VTE) event rates, but there is wide-ranging variation in worldwide practices and international guidelines [5,16]. The guideline by the FMS, which is only applicable to below-knee plaster, states to consider only treating patients with LMWH as pharmacologic thromboprophylaxis if they have a history of VTE or in the case of extensive trauma [17]. Due to the low absolute risk, the high number needed to treat and the possible increased risk of bleeding, this guideline does not recommend thrombosis prophylaxis for patients who are at low-risk. It also states that “high-risk patients cannot currently be identified by a risk score due to a lack of prospective validation” [17]. Neither of these recommendations coincide with the findings of this study, where in most departments, LMWHs are prescribed by default or based on either the Caprini or PADUA score. Notably, the PADUA prediction score is a risk assessment model to determine anticoagulation need for non-surgical hospitalized patients, whereas the Caprini score stratifies the risk of VTE in surgical hospitalised patients [17].

The literature suggests that high vitamin C intake after a wrist fracture reduces the risk to develop complex regional pain syndrome (CRPS) in adults [18]. Consequently, the guideline from the FMS includes the prescription of 500 mg of vitamin C per day orally for 50 days [19]. The guideline does not comment on other types of fractures or the severity of the wrist fracture. A randomized double-blind study showed no difference in outcome between dosages of 500 mg or 1000 mg [18]. Once again, the results of this study do not reflect the general recommendation in either indication or duration.

The splitting of a plaster cast can be used to accommodate anticipated swelling [20]. However, controversy regarding the need for cast splitting remains [21]. Most studies were conducted on paediatric patients and concluded that there is no significant difference between split or bivalve casts relative to circumferential casts. The lack of a guideline could be the origin of the practice variation that is observed in this study.

Studies suggest that suturing a wound with sterile gloves does not differ in terms of infection rates from suturing in an unsterile setting [22]. The guideline by the FMS includes no statements on the use of sterile materials to close a wound, but it does recommend cleaning an acute wound with the use of lukewarm tap water rather than sterile saline or disinfectants [23]. It is clear from the study results that only a minority of EDs follow this recommendation.

A scaphoid fracture can be treated with a short arm cast or a scaphoid cast, although the literature suggests that immobilization of the thumb appears unnecessary [24]. The National Society of Trauma surgery (NVT) recommends a scaphoid cast with immobilisation of the thumb [25].

Due to the low sensitivity of conventional X-ray of the cervical spine, the Dutch Society of Orthopaedics and the FMS recommend a CT scan in all adult patients with an indication for imaging diagnostics of the cervical spine [26]. With the exception of one ED, all departments adhere to this practice. The guideline also states that the literature summary shows that it is unclear whether the NEXUS criteria are sufficiently sensitive to be used as a decision rule. Therefore they advise the Canadian C-Spine Rule (CCR) criteria with the addendum of intoxication (which is part of the Nexus), based on the assessment of the attending physician. This concurs with the results of a large Canadian cohort study which concludes that, for alert and stable patients, the CCR is superior to the NEXUS with regards to sensitivity and specificity for cervical spine injury [27].

The ED application of FICB for patients with hip fractures reduces pain and in-hospital need for opiates [28]. Although it does not deliver complete analgesia for surgery, FICB is recommended for preoperative analgesia [28]. A meta-analysis in 2021 concluded that FICB and femoral nerve block are equally effective for pain control and morphine-sparing in knee and hip surgeries [29].

There are several limitations to this study. The selection of practices that were analysed was based on a pragmatic approach for this study. By studying trauma-related practices with a binary or low-complexity character, it could influence the amount of practice variation that was analysed.

By creating optional questionnaire answers, participants may have been forced to select an answer that best represents their circumstances but does not exactly reflect their situation. To minimize this, the questionnaire had room to elaborate or differentiate answers. However, not all questions had an option that a practice differs between physicians within one ED. It is arguable that physicians who work together in the same department are relatively uniform or similar in their medical policy [30]. Nonetheless, this study merely reveals the variation between hospitals, it does not describe the variation within hospitals. Moreover, this study did not validate to what extent actual treatment on the work floor adhered to registered local guidelines.

Furthermore, this study did not collect data on hospital resources or organisational factors such as pharmaceutical agreements, access to imaging modalities or specific training. Therefore the study could not account for such potentially relevant characteristics in interpretation [15]. Variation in resources and financial means can partially place variation outside the influence of individual physicians or an emergency department [12].

Additionally, the results of this study are not necessarily representative for all EDs in The Netherlands because the minority of EDs that do not employ board-certified emergency physicians were excluded.

Finally, this study mainly compares results with Dutch national guidelines. It would be interesting for a follow-up study to put these results in a broader perspective, including a comparison of (extent of) practice variation within other medical specialties and in other countries, especially in countries were EM is recognised as a medical specialty.

## 5. Conclusions

In conclusion, EDs in The Netherlands show considerable practice variation in treatments among the subjects studied. Additionally, the extent of variation differs per theme. It suggests that variation might not originate from local guidelines but from the wide-ranging offer of (inter)national available guidelines, perhaps combined with ambiguity towards the evidence base or the fact that old habits are hard to eliminate. In order to standardize procedures, improve the quality and reduce the costs of health care, further consultation and consideration is indicated to reduce the amount of the practice variation in the landscape of modern emergency medicine. Based on the results, it is recommended that every ED in The Netherlands assesses the local protocols and frequently updates their content based on the most recent recognised evidence-based guidelines to harmonise practices.

## Figures and Tables

**Table 1:** Results of characteristics of EDs.

Variable		Min.	Mean	Median	Max.
Number of emergency physicians in ED		2	10	10	23
Years emergency physicians present at ED		0	11	11	20
Number of residents in team		0	3	2	16
Number of interns in team		0	9	10	23
Number of patients that visit ED on a yearly basis		10,000	26,615	26,000	50,000
Number of beds in hospital were ED is located		50	415	430	942
Number of beds in emergency department		7	18	17	37
**Variable**	**Academic**	**Top clinical**		**General**	
Hospital category	12%	42%		46%	
**Variable**	**Primary**	**Accommodating**		**None**	
Residency program	69%	19%		12%	
**Variable**	**Level 1**	**Level 2**		**Level 3**	
Trauma level of hospital	21%	50%		29%	

**Table 2:** Results of questionnaire.

**Practice**			Yes		No	PADUA		Other	
**1**	Anticoagulation during immobilization with a lower leg cast		27%		8%	21%		23%	
**Practice**			Yes		No	PADUA		Other	
**2**	Anticoagulation during immobilization with an upper leg cast		75%		0%	8%		17%	
**Practice**			Yes		No	PADUA		Other	
**3**	Anticoagulation during immobilization knee brace/splint		12%		71%	4%		13%	
**Practice**			Min		Mean	Median		Max	
**4**	Age anticoagulation		12		23	18		76	
**Practice**			Uniform			BMI		Other	
**5**	Dose anticoagulant is based on		38%			44%		17%	
**Practice**			Dalteparine		Nadroparine	Tinzaparine		LMWH	
**6**	Anticoagulant medication		35%		60%	2%		4%	
**Practice**			No		Yes	Only after reposition			
**7**	Vitamin C after wrist fracture		58%		27%	15%			
**Practice**			No			Yes			
**8**	Vitamin C other distal fractures		90%			5%			
**Practice**			Min		Mean	Median		Max	
**9**	Age vitamin C		16		27	18		50	
**Practice**			1 dd 500 mg			1 dd 1000 mg		None	
**10**	Dosage of vitamin C		52%			2%		46%	
**Practice**			2 w	4 w	50 d	7 w	2 m	3 m	Im
**11**	For what period is vitamin C prescribed?		2%	2%	31%	4%	2%	8%	6%
**Practice**			No			Yes			
**12**	Standard cleavage of plaster top extremities		68%			31%			
**Practice**			No			Yes			
**13**	Standard cleavage of plaster at the bottom extremities		67%			33%			
**Practice**			CCR			Nexus		Other	
**14**	Trauma protocol cervical spine		17%			69%		13%	
**Practice**			X-CWK			CT-CWK			
**15**	First imaging modality cervical spine		2%			98%			
**Practice**			Sterile materials suturing						
**16**	Sterile gloves		31%						
	Sterile drape		17%						
	Sterile suture set		88%						
	None		12%						
	Other		6%						
**Practice**		Hibicet	Sterile water			Tap water		Other	
**17**	Materials wound cleaning	62%	73%			25%		10%	
**Practice**			Short arm cast			Navicular cast			
**18**	Plaster scaphoid fracture		46%			54%			
**Practice**		Ultrasound FICB	Ultrasound n. femoralis block		Anatomy	Differs per physician		No block	
**19**	Method FICB	40%	12%		0%	19%		29%	

## Data Availability

Not applicable.

## Appendix A

**Table A1:** Content questionnaire practice variation.

	Practice	Data Type	Unit	Scoring System
1	Is anticoagulation given to the patient during the immobilization due to a lower leg cast?	Nominal	-	Yes No Based on PADUA Other, namely:
2	Is anticoagulation given to the patient during the immobilization due to a upper leg cast?	Nominal	-	Yes No Based on PADUA Other, namely:
3	Is anticoagulation given to the patient during the immobilization by means of a knee brace/split?	Nominal	-	Yes No Based on PADUA Other, namely:
4	From what age is anticoagulation given during leg immobilization?	Scale	years	
5	Do you vary the dose of anticoagulant medication that is prescribed based on:	Scale	-	Age BMI Uniform dose Other, namely:
6	Which anticoagulant is given	Nominal	-	
7	Is vitamin C prescribed as a home medication after one wrist fracture?	Nominal	-	Yes No Only after reposition
8	Is vitamin C prescribed as a home medication after other extremity fractures	Nominal	-	Yes, namely: No
9	From what age is vitamin C prescribed?	Scale	years	
10	Which dose of vitamin C is prescribed?	Scale	mg	
11	For which period is vitamin C prescribed	Nominal	-	
12	Is there a standard cleavage of plaster for upper extremity casts?	Nominal	-	Yes No
13	Is there a standard cleavage of plaster for lower extremity casts?	Nominal	-	Yes No
14	Comments on plaster cleavage	-	-	
15	Which protocol is used to release the cervical spine after trauma?	Nominal	-	CCR NEXUS Other, namely:
16	Which form of radiology is the first step in imaging the cervical spine after adult trauma?	Nominal	-	X-CWK CT-CWK
17	Which sterile materials are used as standard in the stitching a wound? (multiple answers possible)	Nominal	-	Sterile gloves Sterile drape Sterile suture set None of the above Other, namely:
18	Which materials are used by default in the cleaning a (stitch) wound? (multiple answers possible)	Nominal	-	Hibiset Sterile Water Tap water Other, namely:
19	What type of plaster is applied for a scaphoid fracture?	Nominal	-	Short arm cast Navicular cast
20	Which method is used for setting a FICB in a patient with a hip fracture?	Nominal	-	Ultrasound-guided Ultrasound-guided femoral block Anatomical landmarks Differs per physician Not performed
21	General comments	n/a	-	

**Table A2:** Content questionnaire characteristics ED.

Variable	Type	Scoring System
Number of Emergency Physicians in ED	Scale	
Years Emergency Physicians are present at ED	Scale	
Number of Residents in team	Scale	
Number of Interns in team	Scale	
Number of patients that visit ED on a yearly basis	Scale	
Number of beds in hospital were ED is located	Scale	
Number of beds at ED	Scale	
Hospital category	Nominal	Academic Top clinical General
Trauma level of hospital	Nominal	Level 1 Level 2 Level 3
Emergency Medicine residency program	Nominal	Primary Accommodating No
